# Integrated transcriptomic and metabolomic analyses reveal biphasic thermal adaptation strategies in *Lavandula angustifolia* under high-temperature stress

**DOI:** 10.7717/peerj.21294

**Published:** 2026-06-17

**Authors:** Kuerban Tusong, Zhen Liu

**Affiliations:** 1 College of Biological Science and Technology, Yili Normal University, Yining, Xinjiang, China; 2 Xinjiang Key Laboratory of Lavender Conservation and Utilization, Yili Normal University, Yining, Xinjiang, China

**Keywords:** *Lavandula angustifolia*, High temperature stress, Transcriptome, Metabolome, Thermotolerance, Metabolic adaptation

## Abstract

**Background:**

*Lavandula angustifolia* is a commercially vital medicinal plant, but its molecular adaptation mechanisms under high-temperature stress (HTS) remain poorly understood. Global warming poses a significant threat to agricultural productivity, necessitating deeper insights into plant thermotolerance strategies for develop climate-resilient crops.

**Methods:**

We employed integrated transcriptomic and metabolomic analyses to investigate the biphasic responses of *L. angustifolia* to HTS. Seedlings were subjected to short-term (1–5 h) and long-term (3–5 days) heat stress at 38 °C. Physiological parameters, including malondialdehyde (MDA) and proline (PRO) levels, were measured. Transcriptomic sequencing and metabolomic profiling were conducted to identify differentially expressed genes (DEGs) and differentially accumulated metabolites (DAMs). Functional enrichment and pathway analyses were performed to elucidate key molecular mechanisms.

**Results:**

Short-term responses featured rapid Ca^2+^-mediated signaling, heat-shock protein activation, and transient oxidative stress, accompanied by osmoprotectant accumulation (*e.g.*, proline and raffinose-family oligosaccharides). Long-term adaptation involved systemic metabolic reorganization, including enhanced citrate cycle (TCA cycle) flux, glyoxylate metabolism, and phenylpropanoid biosynthesis. Key regulators such as aconitate hydratase and galactinol synthase, along with signature metabolites like 2-oxoadipic acid and capric acid, were identified as markers of thermal adaptation. The study reveals a transition from immediate protective responses to sustained metabolic optimization, providing a comprehensive framework for understanding *L. angustifolia*’s thermotolerance. These findings offer actionable targets for breeding heat-resistant cultivars to mitigate climate change impacts on medicinal crop production.

## Introduction

Temperature is a key environmental factor affecting plant growth, development, geographical distribution, quality, and productivity, with the entire plant life cycle influenced by environmental temperatures (Zhou et al., 2023; Ding, Shi & Yang, 2020). Global warming has led to frequent extreme high-temperature events, with projected global average temperature increases of 1.5 °C to 4.8 °C by 2100 (KJKP, 1997). These changes have become major limiting factors for crop productivity and food security (Kang, Khan & Ma, 2009; Makore et al., 2021), causing yield losses, reduced nutritional quality, and threats to agricultural sustainability (Osman et al., 2020; Minoli et al., 2022; Zhang et al., 2023). Elucidating plant heat response mechanisms, identifying heat-resistant gene loci, and clarifying their functions and regulatory networks are essential for addressing food security challenges under global warming.

High temperature stress (HTS) not only impacts plant phenotypes but also disrupts cellular homeostasis, severely affecting growth and development (Kan et al., 2023; Tiwari & Yadav, 2019). Plant responses to HTS can be categorized into three interconnected types: (i) immediate sensing and signal transduction, involving calcium spikes, reactive oxygen species (ROS) bursts, and heat-shock protein (HSP) expression; (ii) physiological and biochemical adjustments, such as osmotic regulation, membrane stabilization, antioxidant activation, and photosynthetic modulation; and (iii) morphological and developmental adaptations, including altered leaf morphology, reduced stomatal density, and accelerated flowering (Wahid et al., 2007; Mittler, Finka & Goloubinoff, 2012; Ohama et al., 2017). Collectively, these responses mitigate heat-induced damage and restore homeostasis through transcriptomic and metabolomic reprogramming (Haider et al., 2021; Li et al., 2018). A deeper understanding of these integrated mechanisms is crucial for improving plant thermotolerance.

Recent advances in temperature stress research have deepened our understanding of molecular regulatory networks for plant temperature sensing and response (Makore et al., 2021). The mechanisms underlying heat stress responses are complex and polygenic, involving signal amplification, transcriptional control, heat shock gene (HSG) expression, and oxidative/osmotic stress tolerance genes (Makore et al., 2021). Research across model and crop species has identified numerous heat-responsive genes, small RNAs, and proteins. For instance, it is known that plants detect heat signals through sensors such as photoreceptor PHYB, ELF3, and PIF7 (Chen, Galli & Gallavotti, 2022). PHYB regulates light/temperature responses, hormone signaling, and development (Devireddy, Liscum & Mittler, 2020), while the evening complex (EC) acts as a thermosensor (Nusinow et al., 2011). Small heat shock proteins (sHSPs) prevent protein misfolding (Yu et al., 2021), and heat shock factors (HSFs) like HSFA1 and HSFA2 regulate thermotolerance and stress memory (Ohama et al., 2017; Friedrich et al., 2021). Other key components include WRKYs, NACs, HSPs, and hormone-related genes (He et al., 2022; Li et al., 2021), highlighting the multi-layered nature of heat adaptation.

While several conserved pathways—such as Ca^2+^ signaling, heat shock protein (HSP) induction, antioxidant defense, and hormone-mediated responses—have been well characterized in model plants (Li et al., 2018), the specific regulatory networks and metabolic adaptations in perennial medicinal species like *Lavandula angustifolia* remain largely unexplored. Investigating these molecular mechanisms can advance genetic thermotolerance research. Integrating physiological, transcriptomic, and metabolomic approaches enables a deeper understanding of stress responses. For example, Zhao et al. (2022) linked thermotolerance in *Paspalum wettsteinii* to heat-responsive genes in energy metabolism and antioxidant systems, while Xie et al. (2023) revealed HSF-mediated purinergic pathway modulation in quinoa. Such integrated analyses bridge genotype-to-phenotype transitions under heat stress.

Lavender (*Lavandula angustifolia* Mill.), a commercially valuable medicinal and aromatic plant (Shahdadi et al., 2017; Gallotte et al., 2020), yields approximately 1,500 tons of essential oil annually from various *Lavandula* species, with true lavender (*L. angustifolia*) contributing 300–500 tons (Wells et al., 2018; Muntean et al., 2016). While France and Bulgaria remain leading producers (Giray, 2019), China’s Xinjiang Yili Valley has emerged as the country’s largest cultivation base (Guo et al., 2020), alongside traditional growing regions in the Mediterranean and Middle East (Batiha et al., 2023; Ez zoubi, Bousta & Farah, 2020). Although extensive research has focused on the essential oil composition, agronomy, and pharmacology of lavender (Turek & Stintzing, 2013; Wells et al., 2018), the molecular mechanisms underlying its resilience to abiotic stresses, particularly high-temperature stress, remain poorly characterized. In plants, exposure to elevated temperatures triggers a suite of molecular and physiological adjustments to mitigate damage and maintain homeostasis (Li et al., 2018). This process, which is called heat stress adaptation, encompasses the dynamic, multi-layered responses—from immediate signaling and protective gene expression to longer-term metabolic reorganization—that collectively enhance plant survival and function under supra-optimal temperatures. In the context of plant responses to high temperature, it is important to distinguish between thermotolerance and heat stress adaptation. Thermotorance refers to the inherent or acquired capacity of a plant to survive and maintain physiological function under high-temperature stress, often involving constitutive and inducible molecular mechanisms (Wahid et al., 2007; Sung et al., 2003). Heat stress adaptation, meanwhile, describes the dynamic process through which a plant adjusts its physiology, metabolism, and gene expression over time to cope with and acclimate to sustained or recurring high-temperature conditions (Li et al., 2018; Ohama et al., 2017). In this study, we investigate both the immediate thermotolerance mechanisms and the longer-term adaptive responses of lavender under progressive heat stress. To decipher these adaptive mechanisms in lavender, we employed an integrative approach combining physiology, transcriptomics, and metabolomics under controlled heat stress. This study delineates the molecular framework of lavender’s heat stress adaptation, identifying key regulatory pathways and potential targets for breeding climate-resilient cultivars.

## Materials & Methods

### Plant material and treatment

The experiment used *L. angustifolia* cultivar ‘French Blue’, a widely cultivated local variety, with seeds obtained from a commercial market in Yining, Xinjiang, China. Healthy, uniform seeds were surface-sterilized by immersion in 75% ethanol (10 mL) in a sterile 50 mL centrifuge tube with gentle shaking (100 rpm) for 5 min, followed by three rinses with sterile distilled water, and then germinated in a greenhouse. After 80 days of growth, seedlings were transferred to an artificial climate chamber (BIC-250; Shanghai Boxun Medical Biological Instruments Co., Ltd., Shanghai, China) for a 7-day acclimation period under controlled conditions (25 °C, 75% relative humidity, 16/8 h light/dark cycle). The acclimated seedlings were divided into two groups: a control group maintained under original conditions and treatment groups subjected to temperature stress at 38 °C for different durations—with heat stress initiated at 14:00 local time (Beijing Time, UTC+8), corresponding to the typical peak daytime temperature period in the Yili Valley, to simulate a natural diurnal heat event—short-term continuous heat (1 h, 3 h, and 5 h, designated as H1, H3, and D1, respectively) and long-term cyclic stress (38 °C/25 °C for 5/19 h daily over 3 and 5 days, designated as D3 and D5, respectively). Corresponding control plants sampled at matched time points are referred to as CK1 (for comparisons with H1, H3, and D1), CK3 (for D3), and CK5 (for D5). Aboveground tissues (stems and leaves) were collected by cutting at the soil surface from six pots for each sample for physiological, transcriptomic, and metabolomic analyses, with samples for transcriptomic and metabolomic analyses being immediately frozen in liquid nitrogen and three biological replicates being prepared per treatment.

### Physiological measurement of lavender

Ground tissue samples (0.1 g) were homogenized in one mL of extraction buffer (Sangon Biotech, Shanghai, China) in an ice bath for malondialdehyde (MDA) content determination. The homogenate was centrifuged at 8,000 × g for 10 min at 4°C. The resulting supernatant was collected for MDA content measurement following the manufacturer’s protocol (Malondialdehyde Assay Kit, Micro Method, Product No. D799762-0100; Sangon Biotech). Briefly, the supernatant was reacted with thiobarbituric acid at 100 °C for 60 min, and the absorbance of the resulting colored product was measured at 532 nm and 600 nm.

For proline (PRO) content analysis, 0.1 g of ground tissue was extracted with one mL of PRO assay kit solution (Sangon Biotech). The samples were homogenized in an ice bath, then incubated in a boiling water bath for 10 min with continuous shaking. After centrifugation at 10,000 × g for 10 min at room temperature, the supernatant was cooled and analyzed according to the kit instructions (Sangon Biotech).

### Transcriptomic sequencing and data analysis

The cDNA library was constructed and sequenced by Novogene (Shanghai, China) using the ABclonal mRNA-seq Lib Prep Kit (ABclonal, Technology, Woburn, MA, USA) following the manufacturer’s protocol. After adaptor ligation, cDNA underwent PCR amplification, and the products were purified with the AMPure XP system. Library quality was verified on an Agilent Bioanalyzer 4150 system before sequencing on the DNBSEQ-RST7-T7 platform to generate 150 bp paired-end reads. Raw FASTQ data were quality-filtered with Fastp (Chen et al., 2018), and high-quality reads were aligned to the lavender genome using HISAT2 (Li et al., 2021; Kim, Langmead & Salzberg, 2015). Differential expression analysis was performed with DESeq2 (Love, Huber & Anders, 2014). All comparisons were defined as each heat-stress treatment group *versus* its corresponding time-matched control group (*i.e.,* H1 *vs* CK1, H3 *vs* CK1, D1 *vs* CK1, D3 *vs* CK3, and D5 *vs* CK5). Prior to analysis, low-expression genes with fewer than 10 reads across all samples were filtered out. Read counts were normalized using the default median-of-ratios method within DESeq2, and differential expression was tested using the Wald test. The resulting *p*-values were adjusted for multiple testing using the Benjamini–Hochberg procedure to control the false discovery rate (FDR). Differentially expressed genes (DEGs) were defined as those with |Log2FoldChange| ≥ 1 and adjusted *p*-value < 0.05 (Wang et al., 2010). The statistical power of our experimental design was assessed using the RNASeqPower R package (v1.44.0). Parameters included: *n* = 3 biological replicates per group (standard for plant transcriptomics), minimum detectable fold-change = 2.0 (matching our DEG threshold), sequencing depth = 30 million reads per sample, and biological coefficient of variation (CV) = 0.4. The calculated power of 52% to detect 2-fold changes at α = 0.05 indicates that while the design is suitable for capturing major transcriptional changes, future studies would benefit from increased replication to enhance sensitivity for subtle changes. Functional annotation utilized BLAST2GO (Gene Ontology (GO) terms) and Kobas (Kyoto Encyclopedia of Genes and Genomes (KEGG) pathways). GO and KEGG enrichment analyses of differentially expressed genes were performed using the clusterProfiler R package (v4.0.5). The background gene set for enrichment tests comprised all expressed genes detected in the transcriptome (Fragments Per Kilobase of transcript per Million mapped reads (FPKM) ≥ 1 in at least one sample). Statistical significance was assessed using the hypergeometric test, and *p*-values were adjusted for multiple comparisons using the Benjamini–Hochberg FDR correction. Terms or pathways with an adjusted *p*-value <0.05 were considered significantly enriched. All visualizations were generated using R (version 3.5.1) and TBtools (Chen et al., 2020).

### Sample extraction and measurements for metabolomic analysis

Frozen samples were ground in liquid nitrogen, and 80 mg aliquots were subjected to metabolite extraction using 1,000 µL of methanol/acetonitrile/water (2:2:1, v/v/v). The mixture was vortexed for 30 s, ultrasonicated at low temperature for 30 min, and stored at −20°C for 10 min (Tang et al., 2022). After centrifugation (14,000 g, 20 min, 4 °C), the supernatant was vacuum-dried and reconstituted in 100 µL of acetonitrile/water (1:1, v/v). Following another centrifugation (14,000 g, 15 min, 4 °C), the supernatant was filtered and analyzed by UPLC-MS/MS (Vanquish UHPLC, Thermo, coupled to an Orbitrap mass spectrometer) through APT-BIO (Shanghai, China).

### Differential metabolite analysis

The sum-normalized data were analyzed using R packages. Multivariate analyses including Pareto-scaled principal component analysis (PCA) and orthogonal partial least-squares discriminant analysis (OPLS-DA) were performed. Metabolite significance was assessed using Student’s *t*-test and variable influence on projection (VIP) values, with significant changes defined as VIP > 1 and *p*-value < 0.05. Pearson’s correlation analysis evaluated relationships between variables. Significantly regulated metabolites were identified based on VIP > 1, *p*-value < 0.05, and fold change (FC) > 1.2 or < 0.83 (Du et al., 2023). Metabolites were annotated using the KEGG pathway database (http://www.kegg.jp/kegg/pathway.html), and pathways containing significantly regulated metabolites underwent metabolite set enrichment analysis (MSEA) with significance determined by hypergeometric test *p*-values (Han et al., 2023). For the purpose of integrated pathway analysis with transcriptomic data, metabolite sets meeting a *p*-value < 0.05 and VIP > 1 were also considered to capture broader coordinated biological trends for subsequent multi-omics integration.

### Integrated analysis of transcriptome and metabolome

Orthogonal partial least squares discriminant analysis was performed by APT-BIO. All DEGs and differentially accumulated metabolites (DAMs) were mapped to KEGG pathways (http://www.kegg.jp/) to identify common pathways. To generate the Venn diagrams depicting overlapping pathways, we included all KEGG pathways that were significantly enriched for DEGs (adjusted *p* < 0.05) and all pathways associated with DAMs (*p* < 0.05 and VIP > 1). The KEGG annotation and enrichment results from both omics datasets were integrated using R (version 3.5.1). Pathways with a *p*-value < 0.05 from this integrated analysis were considered for further biological interpretation. This approach was designed to identify pathways exhibiting coordinated changes at both the transcriptional and metabolic levels, leveraging multi-omics convergence to strengthen biological inference despite inherent differences in statistical power and coverage between the platforms. The complete lists of all KEGG pathways considered in this integrated analysis for each treatment comparison are provided in Table S1.

### RNA extraction and quantitative real-time PCR

Total RNA was extracted using the EASYspin Plant RNA Kit (Aidlab Biotech, Beijing, China) and reverse transcribed with Hifair^®^ III 1st Strand cDNA Synthesis SuperMix (Yesean Biotech, Maryland, USA). For quantitative real-time PCR (qRT-PCR) analysis, 1,000 ng of RNA was reverse transcribed into cDNA and diluted 4-fold. Reactions were performed on an Applied Biosystems^®^ 7500 Fast system using TransStart Top Green qPCR SuperMix (TransGen Biotech, Beijing, China) in 20 µL volumes containing one µL cDNA, 0.4 µL each primer (Table S2), 0.4 µL passive reference dye, 7.8 µL H_2_O, and 10 µL SuperMix. Thermal cycling conditions consisted of 95 °C for 5 min, followed by 40 cycles of 95°C for 15 s, 60 °C for 30 s, and 72 °C for 30 s. The *LaActin* gene (La04G00167) served as internal control, with relative expression calculated using the 2^−ΔΔCt^ method (Livak & Schmittgen, 2001).

### Statistical analysis

Data visualization was performed using Origin 2023. Statistical significance among treatments was determined by one-way ANOVA with Tukey’s test (IBM SPSS 26.0). Heatmaps, GO enrichment scatter plots, and KEGG enrichment scatter plots were generated using APTBIO Cloud Platform, Omicshare tools (https://www.omicshare.com/tools/), and Majorbio Cloud Platform. Final image composition was completed with Adobe Illustrator 2022.

## Results

### Physiological characteristics of lavender under temperature stress

**Figure 1:**
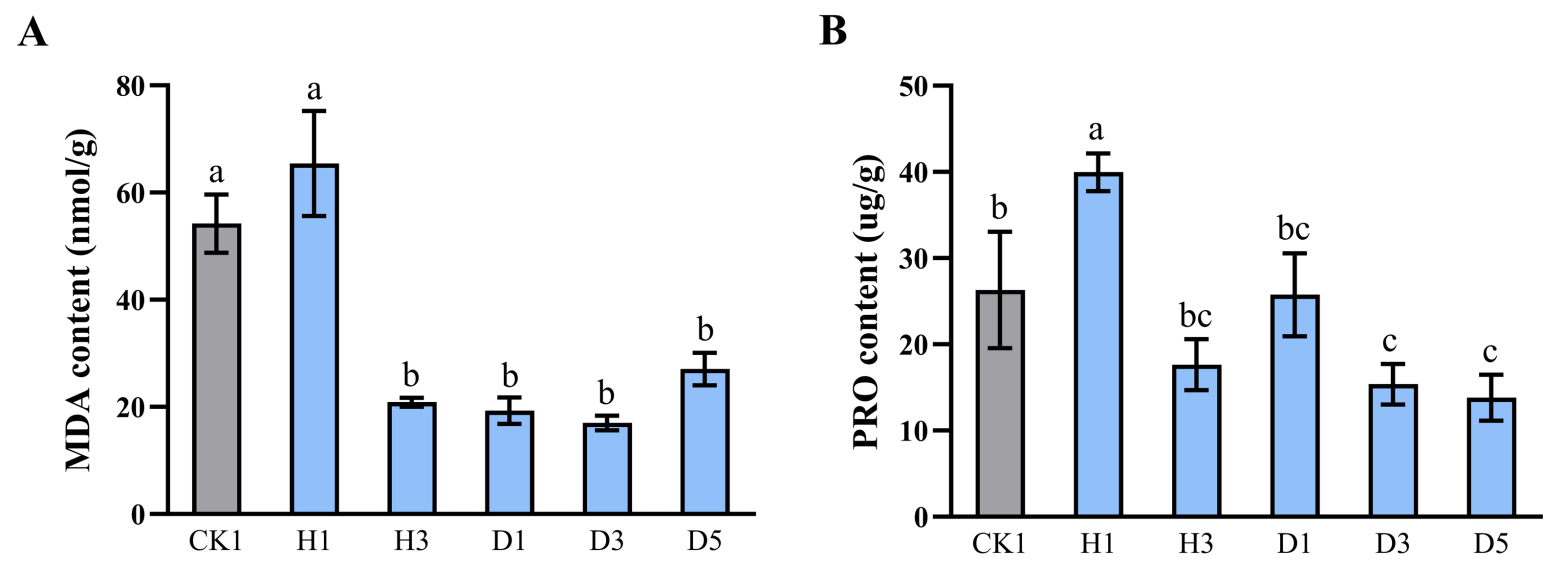
(A) MDA content and (B) PRO content in *L. angustifolia* under different high-temperature stress treatments. a, b and c indicate significant differences (*p* < 0.05).

Under HTS, the MDA content exhibited a mild increase during the H1 (38 °C for 1h) treatment, followed by a gradual decrease over time, reaching significant levels (Fig. 1A). The PRO content also increased significantly during the H1 treatment but subsequently decreased gradually with prolonged HTS exposure, remaining significant except for the H3 (38 °C for 3h) and D1 (38 °C for 5h) treatments (D1 showed a slight increase after H3, but this was not significant compared to CK1) (*p* < 0.05) (Fig. 1B).

### Transcriptomic analysis of lavender during high temperature stress

**Figure 2:**
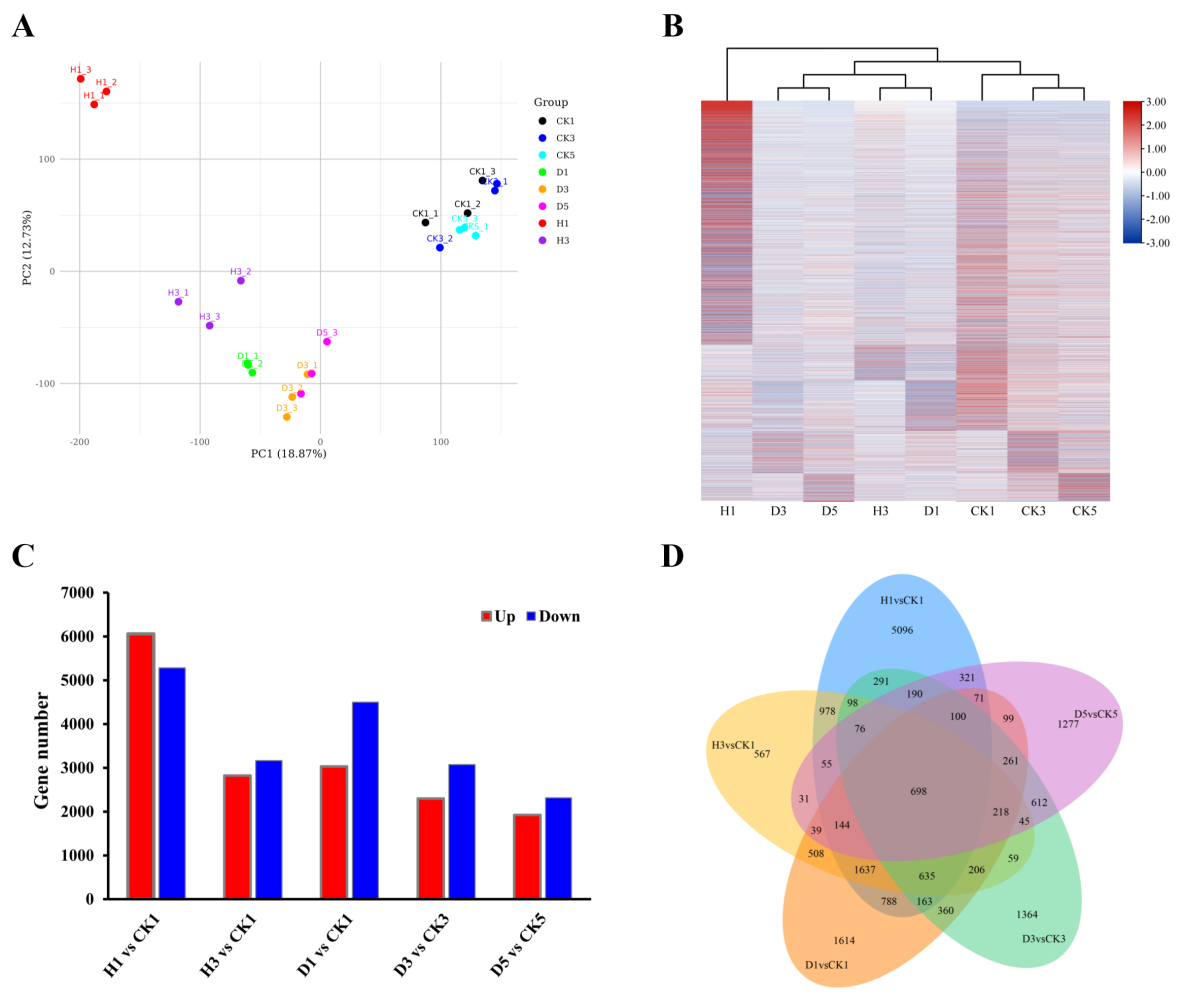
RNA-seq analysis of *L. angustifolia* under high-temperature stress. (A) PCA of RNA-seq samples. (B) Heatmap of DEGs in response to high-temperature stress. (C) Statistical classification of DEGs (up: upregulated genes; down: downregulated genes). (D) Venn diagram of DEGs across different high-temperature-treated samples.

To investigate the gene expression of lavender in response to temperature stress, we conducted RNA-seq analysis. High-quality reads were successfully obtained, with quality control filtering revealing a Q20 value exceeding 98.51% and a Q30 value surpassing 95.71%. The mean GC content was measured at 48.20% (Table S3), confirming that the data met the necessary criteria for subsequent analysis. The trends observed in the PCA of the samples illustrated the degree of variation among them. As shown in Fig. 2A, the PCA results for the RNA-seq samples indicated that both PC1 and PC2 effectively distinguished the control groups (CK1, CK3 (control for D3), CK5 (control for D5)) from the treatment groups (H1, H3, D1, D3 (cyclic 38 °C/25 °C for 3 days), and D5 (cyclic 38 °C/25 °C for 5 days)). Analysis of gene expression abundance revealed distinct transcriptional responses between short-term (H3, D1) and long-term (D3, D5) heat stress. Furthermore, the initial heat shock response (H1) exhibited a markedly divergent pattern from both short- and long-term stress conditions (Fig. 2B). In total, 11,341, 5,994, 7,541, 5,376 and 4,237 DEGs were identified in lavender during H1, H3, D1, D3, and D5 stress, respectively (Tables S4–S8). Among them, in the H1 *vs* CK1, H3 *vs* CK1, D1 *vs* CK1, D3 *vs* CK3, and D5 *vs* CK5 comparisons, respectively, 6,059, 2,824, 3,037, 2,300 and 1,921 genes were up-regulated, while 5,282, 3,170, 4,504, 3,076 and 2,316 genes were down-regulated (Fig. 2C). In addition, 5,096, 567, 1,614, 1,364 and 1,277 genes were specifically differentially expressed under H1, H3, D1, D3, and D5 treatment, respectively, while 698 genes were differentially expressed after all treatments (Fig. 2D). Collectively, these data demonstrate that the highest number of genes were differentially expressed under H1 stress compared with other treatment groups, and up-regulated genes outnumbered down-regulated ones. Furthermore, the number of DEGs decreased with the extension of treatment duration in both short-term (H1 H3, and D1) and long-term (D3 and D5) treatments.

**Figure 3:**
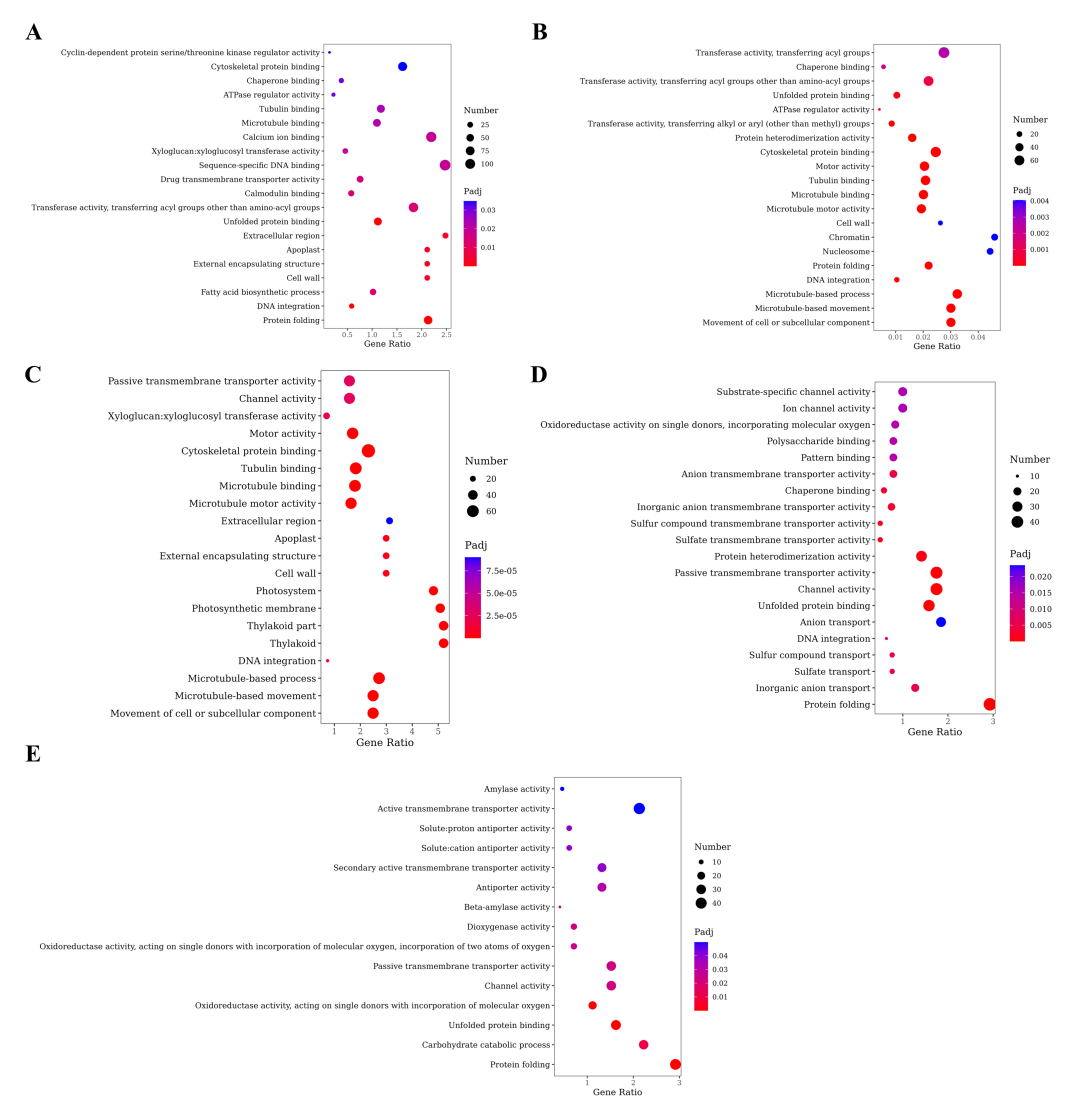
GO enrichment analysis of DEGs responsive to different high-temperature stress treatments. (A–E) Results under H1, H3, D1, D3 and D5 stress conditions, respectively. Dot size represents the number of enriched genes, and color indicates the adjusted *p*-value (*p*_*adj*_).

To further investigate the functional characteristics of DEGs under different temperature stress conditions, we performed GO enrichment analysis. The analysis encompassed all DEGs (both up- and down-regulated) from five comparative groups: H1 *vs* CK1, H3 *vs* CK1, D1 *vs* CK1, D3 *vs* CK3, and D5 *vs* CK5. These genes were classified into 81 GO terms spanning three primary categories based on functional annotations: biological process (BP), molecular function (MF), and cellular component (CC) (Fig. 3). Notably, the MF and BP categories contained the predominant proportion of DEGs. Within the MF classification, the majority of DEGs were associated with “binding” and “transporter activity” subcategories. Detailed analysis revealed significant enrichment of DEGs in specific biological processes under different stress conditions: H1, H3, D1, D3, and D5 treatments showed prominent involvement in sequence-specific DNA binding, calcium ion binding, cytoskeletal protein binding, transferase activity (particularly acyl group transfer), passive transmembrane transporter activity, channel activity, unfolded protein binding, protein folding, and carbohydrate catabolic processes. Of particular interest, microtubule-based processes emerged as the predominant functional category specifically under H3 and D1 stress conditions.

**Figure 4:**
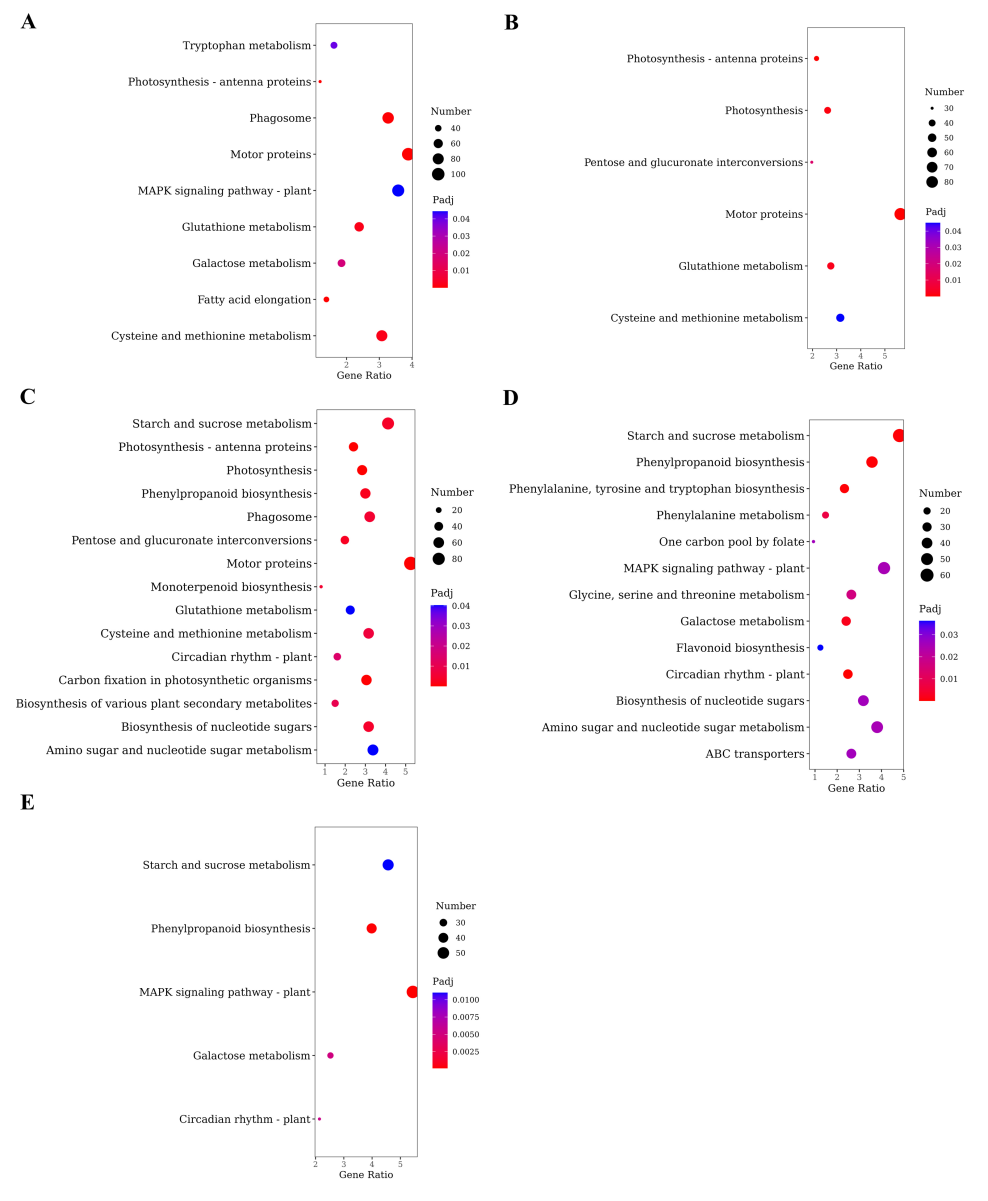
KEGG pathway enrichment analysis of DEGs under different temperature stress conditions. (A–E) Results for H1, H3, D1, D3 and D5 stress treatments, respectively. Bubble size corresponds to the gene count, while color intensity represents the adjusted *p*-value (*p*_*adj*_).

KEGG enrichment analysis of DEGs across five comparison groups was conducted, identifying 10, 6, 15, 13, and 5 KEGG pathways in the H1 *vs* CK1, H3 *vs* CK1, D1 *vs* CK1, D3 *vs* CK3, and D5 *vs* CK5 groups, respectively. In the H1 *vs* CK1 comparison, significant enrichment was observed in pathways related to motor proteins, fatty acid elongation, photosynthesis-antenna proteins, *etc*. (Fig. 4A). The H3 *vs* CK1 comparison revealed significant enrichment for pathways associated with motor proteins, photosynthesis-antenna proteins, photosynthesis, *etc*. (Fig. 4B). For the D1 *vs* CK1 comparison, pathways related to photosynthesis-antenna proteins, motor proteins, photosynthesis, *etc*. were significantly enriched (Fig. 4C). In the D3 *vs* CK3 comparison, significant enrichment was found in pathways related to circadian rhythm-plant, phenylpropanoid biosynthesis, phenylalanine, tyrosine, and tryptophan biosynthesis, *etc*. (Fig. 4D). Finally, in the D5 *vs* CK5 comparison, the pathways of MAPK signaling pathway-plant, phenylpropanoid biosynthesis, galactose metabolism, *etc*. were significantly enriched (Fig. 4E).

### Metabolomic analysis of lavender during high temperature stress

**Figure 5:**
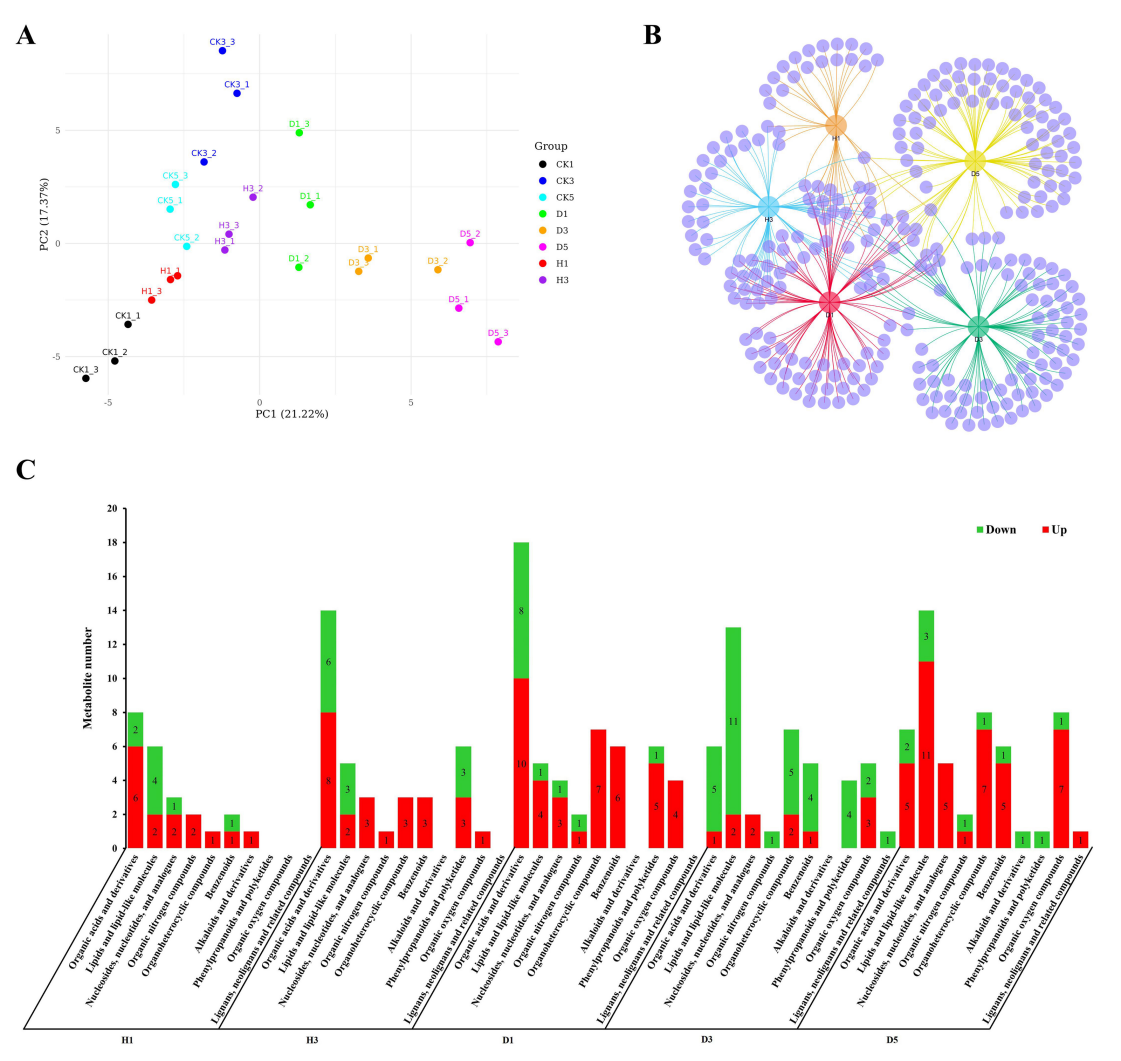
Metabolic profiling of *L. angustifolia* under different high-temperature stress conditions. (A) PCA of metabolic profiles. (B) Venn diagram illustrating shared and unique DAMs among H1, H3, D1, D3 and D5 stress treatments. (C) Statistical analysis of DAMs. Red and green colors denote up-regulated and down-regulated metabolites, respectively, with bar heights indicating metabolite counts.

Metabolomic analysis was conducted using both positive (POS) and negative (NEG) ion modes to maximize metabolite coverage and detection sensitivity. The PCA analysis demonstrated excellent reproducibility among biological replicates, with distinct separation patterns observed: PC1 effectively discriminated between D1 and CK1, D3 and CK3, as well as D5 and CK5 samples, while PC2 differentiated H1 and H3 treatments from CK1 controls (Fig. 5A). Comprehensive non-targeted metabolomic profiling identified 1,289 metabolites in total, comprising 797 detected in positive ion mode and 492 in negative ion mode. Systematic classification revealed that 53.53% of these compounds belonged to ten major categories (Table S11): with lipids and lipid-like molecules (30.23%), phenylpropanoids and polyketides (16.86%), and organic acids and derivatives (12.65%) being the most abundant.

Differential metabolite analysis identified distinct sets of DAMs across the experimental comparisons: H1_vs_CK1 (37 DAMs: 25 up, 12 down), H3_vs_CK1 (66: 41 up, 25 down), D1_vs_CK1 (90: 64 up, 26 down), D3_vs_CK3 (91: 21 up, 70 down), and D5_vs_CK5 (88: 61 up, 27 down). Notably, 1-Methyladenosine was consistently up-regulated under all stress treatments (Table S17). These DAMs were primarily distributed within the major metabolite categories identified above. Notably, lipids and lipid-like molecules, organic acids and derivatives, and phenylpropanoids and polyketides constituted the most substantially altered classes under high-temperature stress (Fig. 5C). Furthermore, quantitative analysis revealed that up-regulated metabolites generally outnumbered down-regulated ones across treatments, with the exception of the D3 condition.

KEGG pathway enrichment analysis of metabolomic data revealed treatment-specific patterns, with distinctions made between nominally enriched and statistically significant pathways (Tables S12–S16). H1 stress showed enrichment (*p*-value < 0.05) in three metabolic pathways: 2-Oxocarboxylic acid metabolism, C5-Branched dibasic acid metabolism, and the citrate cycle (TCA cycle). Glyoxylate and dicarboxylate metabolism also displayed a notable change trend. It is noteworthy that the TCA cycle and Glyoxylate and dicarboxylate metabolism were also significantly enriched (adjusted *p* < 0.05) in the parallel transcriptomic analysis (see Integrated analysis), providing stronger multi-omics support for their activation. H3 stress specifically showed nominal enrichment for C5-Branched dibasic acid metabolism, with associated metabolite changes also observed in Glyoxylate and dicarboxylate metabolism, Taurine and hypotaurine metabolism, and the TCA cycle. For D1 and D3 stresses, while no pathways met the formal significance threshold after multiple testing correction, we observed metabolite associations suggestive of alterations in Sulfur metabolism, Lysine degradation, Valine/Leucine/Isoleucine degradation, Fructose/Mannose metabolism, and Glucosinolate biosynthesis. D5 stress resulted in the nominal enrichment (*p* < 0.05) of Starch and Sucrose metabolism. Several other pathways, including Steroid biosynthesis, Cyanoamino acid metabolism, and Fatty acid biosynthesis, also showed enrichment (*p* < 0.05). Additional pathways with nominal enrichment signals included Cysteine/Methionine metabolism, Carbapenem biosynthesis, Nitrogen metabolism, and Plant hormone signal transduction. The convergence of these metabolic alteration trends with evidence from the transcriptomic dataset (see Integrated analysis) reinforces their potential biological relevance. These shifts in metabolite profiles, including changes in farnesyl pyrophosphate, L-glutamic acid, *α*, *α*-trehalose, and various organic acids (Fig. S1), collectively indicate that *L. angustifolia* undergoes extensive metabolic reprogramming in response to heat stress. This reprogramming involves the coordinated modulation of multiple pathways, including those related to secondary metabolite biosynthesis.

### Integrated pathway analysis of transcriptomic and metabolite expression levels

**Figure 6:**
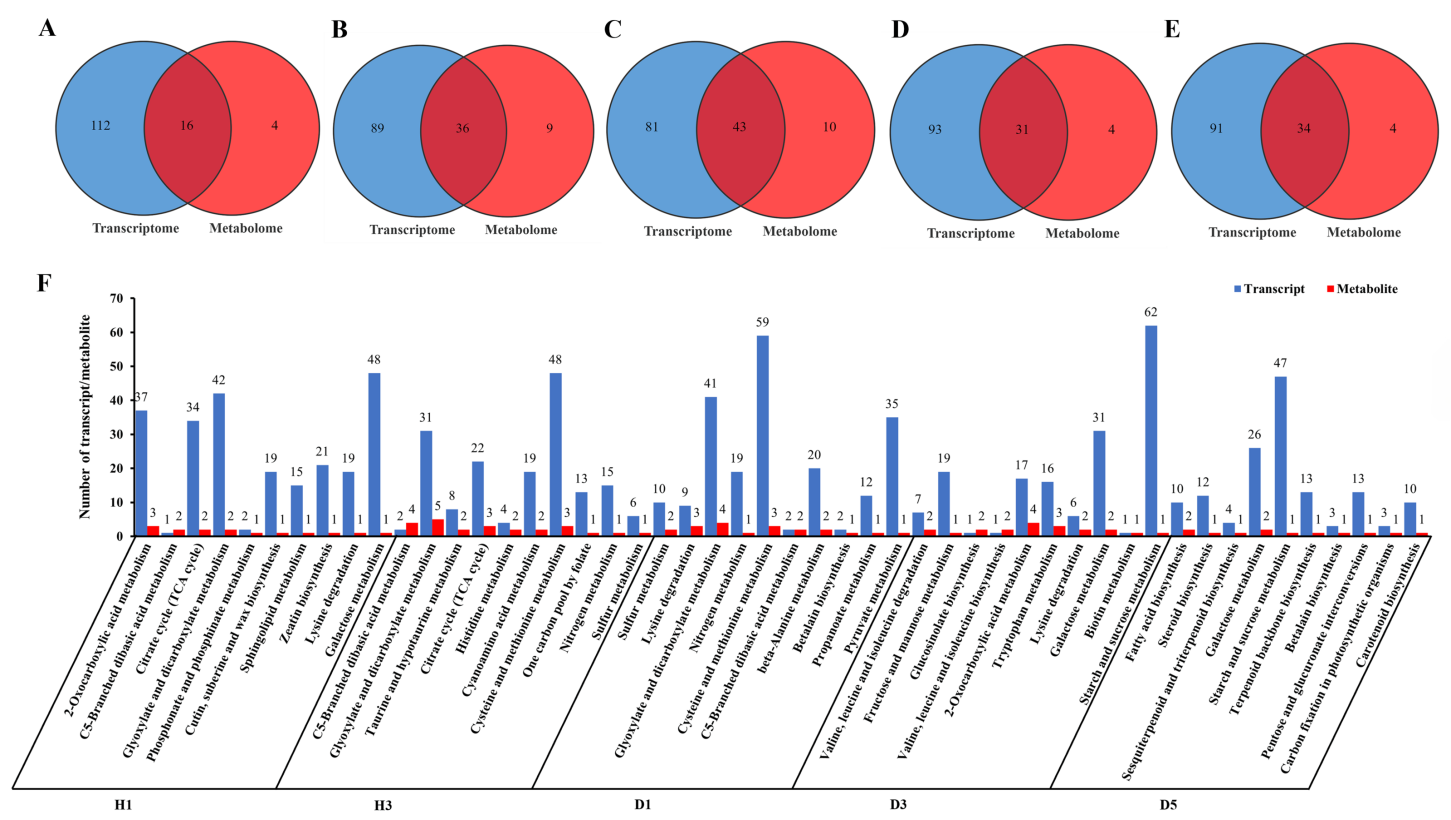
Integrated analysis of transcriptomic and metabolomic responses in *L. angustifolia* under temperature stress. (A–E) Venn diagrams showing overlapping pathways containing both DEGs and DAMs under H1, H3, D1, D3 and D5 stress conditions, respectively. (F) Top 10 significantly enriched KEGG pathways with the highest numbers of co-occurring DEGs and DAMs across all stress treatments. The *x*-axis represents shared pathways, while the *y*-axis displays the combined counts of associated genes and metabolites.

To comprehensively understand the molecular mechanisms underlying lavender’s response to temperature stress, we performed an integrated analysis of transcriptomic and metabolomic data to identify pathways showing coordinated changes in both gene expression and metabolite accumulation. Our analysis revealed that 16, 36, 43, 31, and 34 KEGG pathways were commonly affected in both datasets under H1, H3, D1, D3, and D5 stress conditions, respectively (Figs. 6A–6E). The complete lists of all pathways analyzed for each treatment, including those unique to each omics dataset, are provided in Table S3. Figure 6F presents the top 10 most significantly impacted pathways based on combined gene and metabolite changes. Under H1 stress conditions, the most prominently altered pathways included 2-Oxocarboxylic acid metabolism, C5-Branched dibasic acid metabolism, the TCA cycle, and Glyoxylate and dicarboxylate metabolism. The H3 stress treatment showed similar but less extensive pathway perturbations, with significant enrichment observed in C5-Branched dibasic acid metabolism, Glyoxylate and dicarboxylate metabolism, and the TCA cycle. During D1 stress exposure, we detected coordinated changes in Sulfur metabolism, Lysine degradation, and Glyoxylate and dicarboxylate metabolism pathways, while the C5-Branched dibasic acid metabolism pathway continued to show differential activity. The D3 stress condition specifically enriched the Valine, Leucine, and Isoleucine degradation pathway, with persistent alterations in 2-Oxocarboxylic acid metabolism, Lysine degradation, and Galactose metabolism. Finally, under D5 stress, Fatty acid biosynthesis emerged as one of the most significantly enriched pathways, accompanied by continued changes in Galactose metabolism and Starch and sucrose metabolism pathways. This temporal progression of pathway alterations demonstrates the dynamic nature of lavender’s metabolic adaptation to prolonged temperature stress.

### Analysis of DEGs and DAMs under different temperature stress

**Figure 7:**
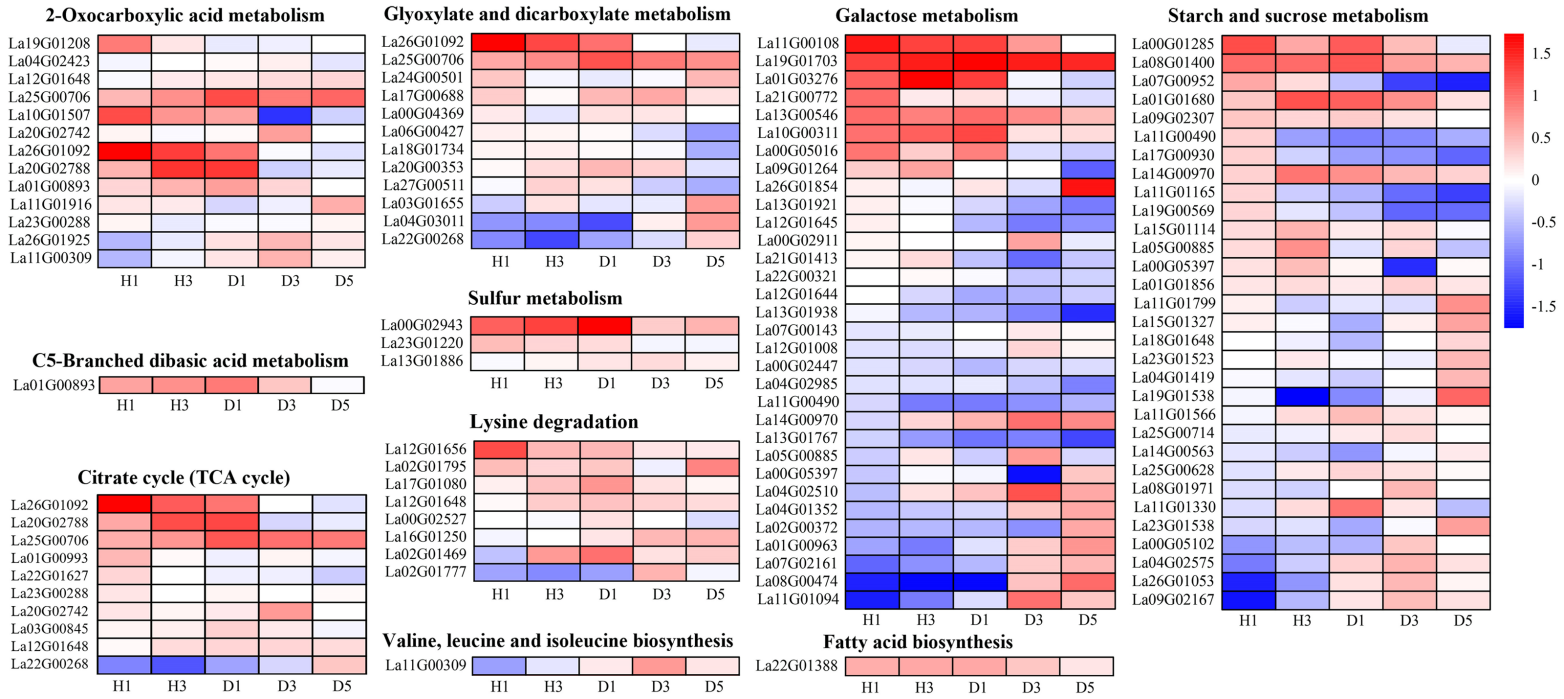
Heatmap analysis of DEGs associated with KEGG pathways under different temperature stress conditions (H1, H3, D1, D3, and D5). Gene expression levels are represented as log2FC values relative to control conditions.

Based on the integrated analysis of transcriptomic and metabolomic data, the top 10 KEGG pathways were evaluated under stress conditions H1, H3, D1, D3, and D5 (Fig. 6F). A total of 491 DEGs were identified, all of which were up-regulated in at least one of these high-temperature (HT) treatment conditions. Specifically, under H1, there were 123 up-regulated DEGs and 163 down-regulated DEGs; under H3, 94 DEGs were up-regulated and 197 were down-regulated; under D1, 80 DEGs were up-regulated and 229 were down-regulated; under D3, 66 DEGs were up-regulated and 217 were down-regulated; and under D5, 75 DEGs were up-regulated and 175 were down-regulated. The up-regulated genes associated with the most significantly affected pathways included: 2-Oxocarboxylic acid metabolism (13 genes), C5-branched dibasic acid metabolism (one gene), citrate cycle (10 genes), glyoxylate and dicarboxylate metabolism (12 genes), sulfur metabolism (three genes), lysine degradation (eight genes), valine, leucine, and isoleucine degradation (one gene), galactose metabolism (32 genes), fatty acid biosynthesis (one gene), and starch and sucrose metabolism (31 genes) under various HTS conditions, as illustrated in Fig. 7.

**Figure 8:**
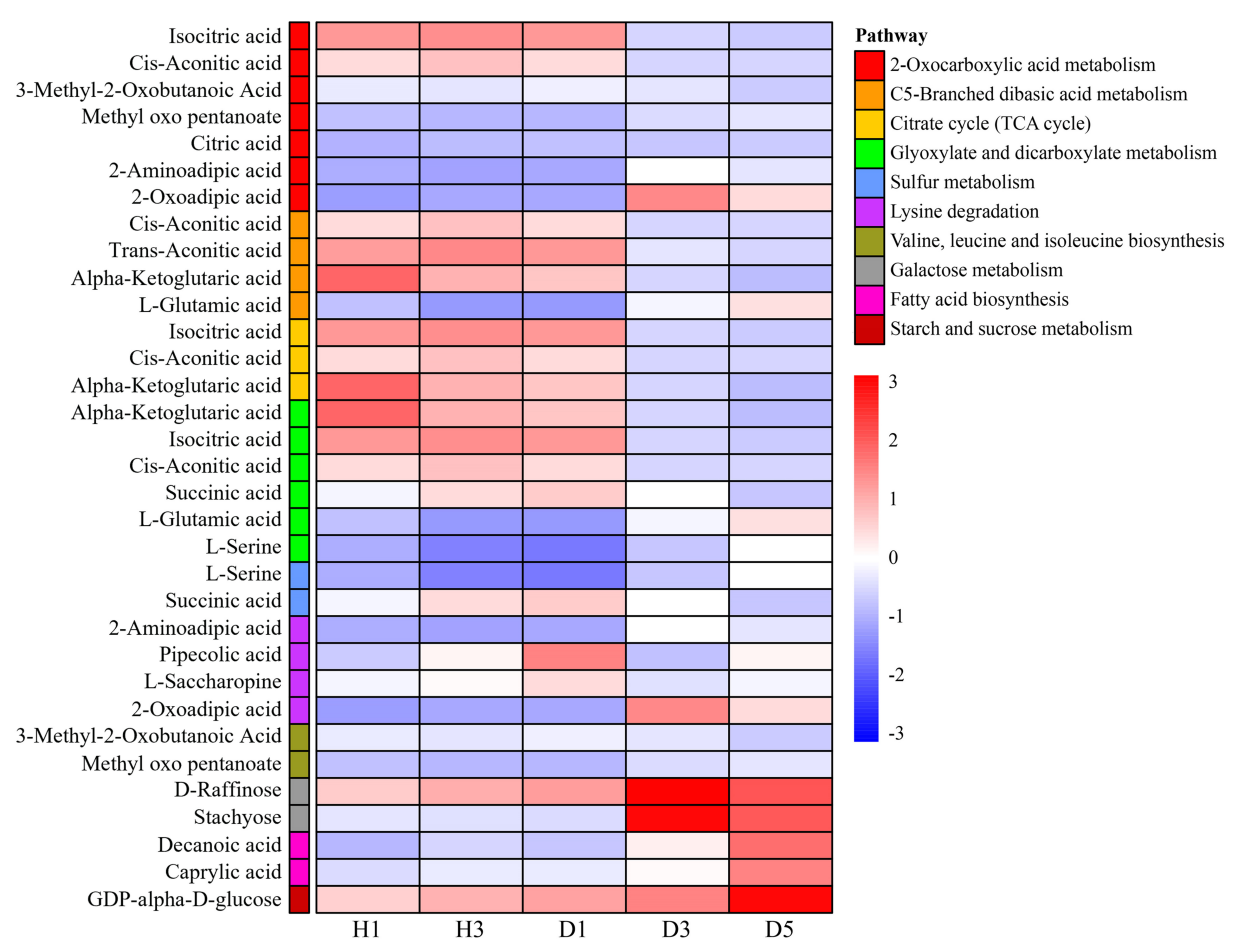
Heatmap analysis of differentially accumulated metabolites under H1, H3, D1, D3, and D5 stress treatments. log2FC values were used to construct the heatmap.

In the DAMs, seven were enriched in 2-Oxocarboxylic acid metabolism, while four were associated with C5-branched dibasic acid metabolism. Notably, three of these metabolites significantly accumulated during short periods of HTS (H1, H3, and D1). Additionally, three DAMs were linked to the TCA cycle, all of which exhibited significant accumulation during the same short periods of HTS. Six DAMs were categorized under glyoxylate and dicarboxylate metabolism, with four showing significant accumulation during these HTS periods. Furthermore, two DAMs were differentially accumulated in sulfur metabolism. Four DAMs were associated with lysine degradation, all demonstrating moderate levels of accumulation after five days of HT treatment (D5). Two DAMs were linked to the biosynthesis of valine, leucine, and isoleucine. Similarly, two DAMs were assigned to galactose metabolism, both significantly accumulating during the extended HTS periods (D3 and D5). A comparable trend was observed in fatty acid biosynthesis, where two DAMs reached their peak accumulation at D5. Lastly, one DAM was associated with starch and sucrose metabolism, exhibiting high accumulation across all stress conditions and peaking with the duration of HTS (Fig. 8).

### qRT-PCR verification of RNA-seq data

**Figure 9:**
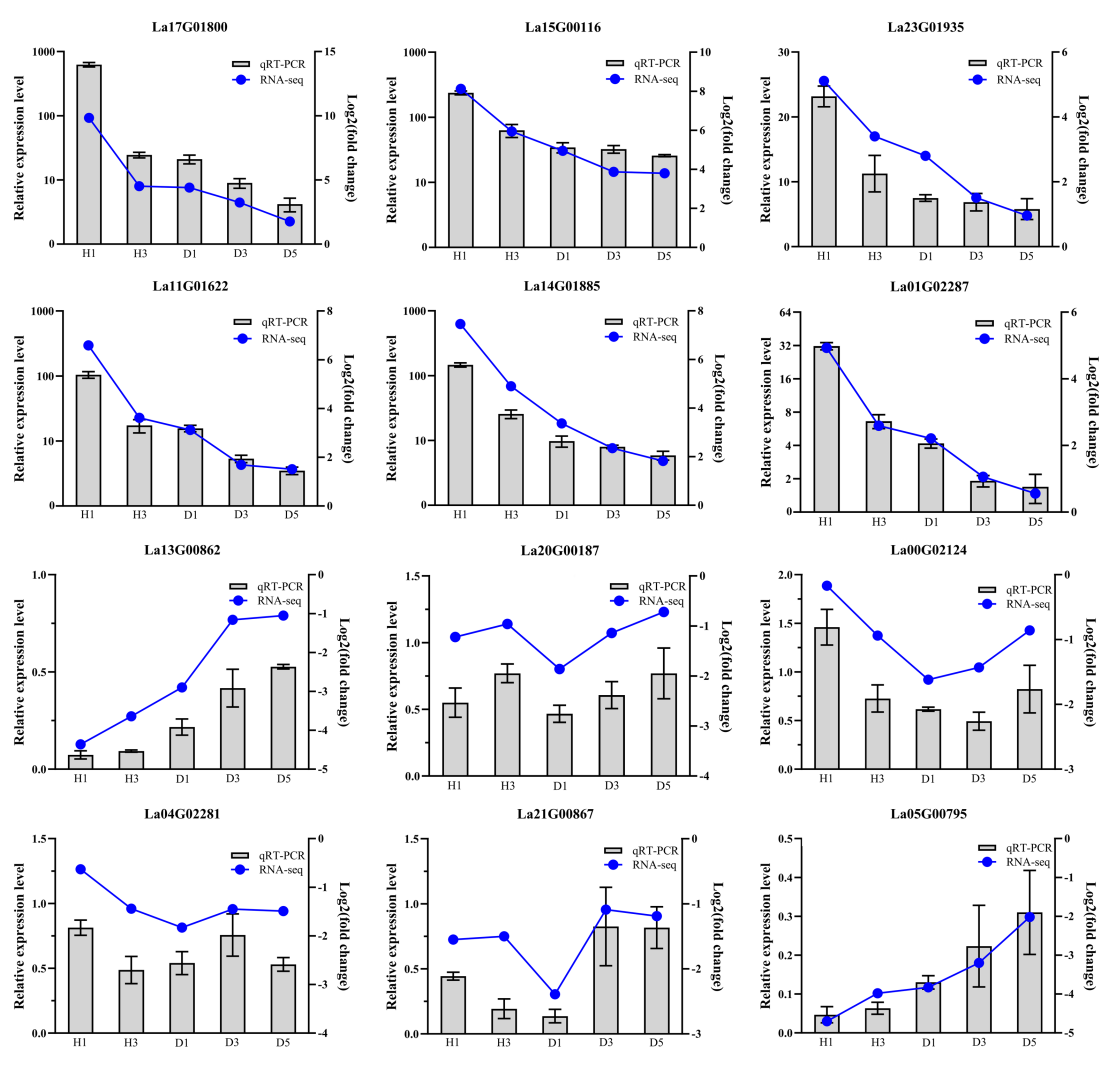
Comparison of RNA-seq and qRT-PCR results for selected genes. Gray bars display RNA-seq log2FC values, with blue line indicating qRT-PCR results (2 − ΔΔCt method).

To validate the RNA-seq results, we performed qPCR analysis on 16 randomly selected DEGs. Among these, six genes (La17G01800, La15G00116, La23G01935, La11G01622, La14G01885, and La01G02287) showed up-regulation under HTS, with relative expression levels >1. These genes exhibited peak expression during H1 treatment, followed by a gradual decline through H3, D1, D3, and D5 treatments. Conversely, six other genes (La13G00862, La20G00187, La00G02124, La04G02281, La21G00867, and La05G00795) were down-regulated under HTS (relative expression < 1). Notably, La13G00862 and La05G00795 displayed a progressive increase in expression with prolonged heat treatment duration. The remaining four down-regulated genes (La20G00187, La00G02124, La04G02281, and La21G00867) showed decreased expression during initial heat treatments (H1-H3-D1) but exhibited recovery at D3 and D5. The strong correlation between RNA-seq and qPCR results (Fig. 9) confirms the reliability of our transcriptome data.

## Discussion

Temperature stands as one of the most critical environmental factors governing plant growth and distribution. While HTS-induced oxidative damage (*e.g.*, ROS accumulation, lipid peroxidation) has been well characterized in model species (Nosaka & Nosaka, 2017; Djanaguiraman, Prasad & Seppanen, 2010), how perennial medicinal plants like *L. angustifolia* coordinate rapid protection with sustained metabolic acclimation remains poorly understood. Our integrated analysis reveals a distinct temporal pattern in *L. angustifolia* under high temperature conditions: MDA content exhibited an initial non-significant increase during early HT exposure, followed by a gradual decrease as plants acclimated to prolonged thermal stress. Notably, D5 samples displayed a marginal (albeit non-significant) elevation in MDA levels after five days of continuous HT treatment. This biphasic response pattern may be attributed to *L. angustifolia*’s capacity to activate antioxidant defenses at 38 °C, enabling both scavenging of excess ROS and restoration of redox homeostasis—a temperature-specific adaptive mechanism that aligns with the species’ evolutionary thermal tolerance threshold.

The accumulation of osmoprotectants, particularly primary metabolites like proline, represents a crucial adaptive mechanism in plants under HTS (Sakamoto & Murata, 2000). These compounds mechanistically participate in osmotic adjustment by enhancing protein stability and stabilizing membrane bilayer structures (Sung et al., 2003). Our experimental results demonstrated dynamic proline regulation in *L. angustifolia* under thermal challenge: Proline content peaked significantly (*p* < 0.05) following 1-hour exposure at 38°C (short-term stress), subsequently declined at the 3-hour time point, showed transient resurgence after 5 h, and progressively decreased during prolonged HT exposure (3–5 days). This pattern is similar to findings in *Apium graveolens* under comparable thermal regimes (Li et al., 2022a; Li et al., 2022b). The observed metabolic trajectory suggests an acute-phase osmoprotection strategy where initial proline accumulation counteracts immediate thermal damage, followed by metabolic optimization as plants acclimate. The gradual decline under chronic stress likely reflects energy reallocation toward sustained thermotolerance mechanisms rather than transient osmolyte production.

The DEGs analysis revealed that the acute phase of HT stress triggered rapid transcriptional reprogramming in lavender, characterized by predominant up-regulation of DEGs following H1 treatment. However, prolonged stress duration resulted in progressive DEG reduction, concomitant with a shift toward down-regulated gene dominance, suggesting metabolic prioritization during acclimation. Notably, this biphasic regulation pattern—initial up-regulation followed by suppression—was conserved in taxonomically distinct celery under chronic HT exposure (Li et al., 2022a; Li et al., 2022b), implying potential evolutionary convergence in plant thermotolerance strategies.

GO enrichment analysis demonstrated that upon initial exposure to heat shock, lavender exhibited significant enrichment of genes associated with sequence-specific DNA binding and calcium ion-binding activities, with both functional categories showing differential regulation. Notably, the enrichment of sequence-specific DNA binding under HS was also observed in celery subjected to HT treatment for one hour (Li et al., 2022a; Li et al., 2022b). This finding aligns with previous studies demonstrating that calcium (Ca^2+^) signaling is among the most rapidly activated pathways in plants under HT stress (Li et al., 2018). Specifically, the upregulation of calcium ion-binding related genes in lavender further corroborates the pivotal role of Ca^2^^+^-mediated signaling in early plant responses to thermal challenges. Extended heat treatment (3–5 h) in lavender induced differential regulation of genes associated with microtubule-based processes and cytoskeletal protein binding. This observation aligns with previous findings in other plant species under thermal stress. When tomato plants were subjected to 4-hour heat treatment, the DEGs similarly showed functional enrichment in binding-related activities (Ding et al., 2020). A parallel pattern emerged in heat-stressed celery exposed to 6-hour thermal challenge, where structural molecular activity represented one of the most significantly enriched categories among DEGs (Li et al., 2022a; Li et al., 2022b). Following repeated heat treatment (3–5 days), lavender exhibited significant enrichment of DEGs associated with transmembrane transporter activity, channel activity, unfolded protein binding, protein folding, and carbohydrate catabolic processes. This pattern aligns with the well-documented plant stress response mechanism wherein heat exposure induces protein misfolding and denaturation, thereby triggering cytoplasmic protein quality control pathways such as the unfolded protein response (Li et al., 2018). Concurrently, lavender likely mobilizes carbohydrate catabolism and transmembrane transport systems to maintain solute homeostasis and cellular osmoregulation under thermal stress conditions.

KEGG pathway enrichment analysis revealed that tryptophan metabolism was significantly enriched in lavender under 1-hour HT treatment, consistent with previous findings in celery (Li et al., 2022a; Li et al., 2022b), while motor protein-associated pathways, fatty acid elongation, and photosynthesis-antenna proteins emerged as the most prominently enriched categories. Following 3-hour HT exposure, photosynthesis-antenna proteins constituted the most significantly enriched pathway in lavender, paralleling responses in *Rosa hybrida* under similar conditions (Wang et al., 2024), with concurrent enrichment of motor proteins and photosynthesis-related pathways. Extended 5-hour HT treatment revealed predominant DEG enrichment in lavender’s photosynthesis-antenna proteins, consistent with *Rosa hybrida* subjected to 6-hour thermal stress (Wang et al., 2024), while motor protein pathways and core photosynthetic processes maintained significant enrichment throughout this duration. Repeated 5-hour daily HT treatments over three/five consecutive days induced significant phenylpropanoid biosynthesis pathway enrichment in lavender, mirroring Red Pitaya responses (Jiao et al., 2021), accompanied by co-enrichment of phenylalanine/tyrosine/tryptophan biosynthesis pathways—the latter coinciding with alfalfa’s phenylalanine metabolic activation under comparable stress (Zhou et al., 2024). Conserved MAPK signaling pathway enrichment in lavender, alfalfa (Zhou et al., 2024), and Red Pitaya (Jiao et al., 2021) implies its cross-species thermotolerance role, with additional prominence of circadian rhythm-plant, starch/sucrose metabolism, and galactose metabolism pathways under these experimental regimes.

Metabolomic analysis showed that lavender under 1-hour HT stress (heat shock) accumulated significant amounts of organic acids and their derivatives, lipids/lipid-like molecules, nucleosides/nucleotides (and analogues), and organic nitrogen compounds. Extended heat acclimation produced dynamic metabolic changes: after 3 h of HT exposure, organoheterocyclic compounds, benzenoids, phenylpropanoids and polyketides became dominant metabolites while maintaining accumulation of organic acids and their derivatives, lipids/lipid-like molecules, nucleosides/nucleotides (and analogues), and organic nitrogen compounds. With 5 h of continuous stress, organic oxygen compounds were additionally observed among the prominently accumulated compounds. Repeated 5-hour daily HT stress for 3 days led to preferential accumulation of lipids and lipid-like molecules, nucleosides/nucleotides/analogues, organoheterocyclic compounds, and organic oxygen compounds. Most significantly, 5-hour daily HT stress for 5 days (extended acclimation) caused marked cumulative increases in organic acids and derivatives, lipids and lipid-like molecules, nucleosides/nucleotides/analogues, organoheterocyclic compounds, benzenoids, and organic oxygen compounds. This progressive metabolite accumulation pattern reveals lavender’s phased adaptation strategy to thermal stress, where initial shock responses (organic acid/nucleotide accumulation) systematically transition to complex secondary metabolite production (including organoheterocyclics, benzenoids and polyketides) during prolonged acclimation.

Integrated transcriptomic and metabolomic pathway enrichment analysis revealed that lavender initially responded to HT stress by enriching DEGs and DAMs in 2-oxocarboxylic acid metabolism, C5-branched dibasic acid metabolism, the TCA cycle, and glyoxylate and dicarboxylate metabolism, with C5-branched dibasic acid metabolism, glyoxylate and dicarboxylate metabolism, and the TCA cycle remaining enriched after 3 h of treatment, while sulfur metabolism and lysine degradation emerged as newly enriched pathways by 5 h, mirroring findings in HT-exposed celery where sulfur metabolism was rapidly induced and lysine degradation pathways were significantly altered after prolonged exposure (Li et al., 2022a; Li et al., 2022b), suggesting these pathways represent conserved early thermal stress responses across species; extended HT treatment of lavender for three days further enriched valine, leucine and isoleucine degradation while maintaining perturbations in C5-branched dibasic acid metabolism, lysine degradation and galactose metabolism, with five-day exposure additionally inducing fatty acid biosynthesis while sustaining alterations in galactose metabolism and starch/sucrose metabolism, paralleling observations in other species where prolonged HS similarly modulates amino acid biosynthesis (Li et al., 2022a; Li et al., 2022b) and glycerolipid metabolism (Zhou et al., 2024), collectively indicating that lavender’s thermal adaptation involves both conserved mechanisms (amino acid and fatty acid metabolism) and species-specific adjustments (C5-branched dibasic acid and carbohydrate metabolism pathways).

The transcriptomic and metabolome pathway co-enrichment analysis revealed that the 2-oxocarboxylic acid metabolism pathway was significantly enriched in short-term HT-stressed lavender samples, with 13 genes showing upregulation under at least one HT treatment condition—particularly during early stress exposure (H1, H3, and D1). The most markedly upregulated genes encoded key metabolic enzymes, including aspartate aminotransferase (La19G01208), which is crucial for amino acid metabolism and Krebs cycle-related organic acid biosynthesis (Schultz et al., 1998); aconitate hydratase (La25G00706 and La26G01092), catalyzing the isomerization of citrate to isocitrate *via* cis-aconitate; UDP-glycosyltransferase (La10G01507), involved in stress-responsive glucosinolate biosynthesis (Grubb et al., 2004); pyruvate dehydrogenase (La20G02788), which drives the conversion of pyruvate to acetyl-CoA (Korotchkina, Sidhu & Patel, 2006); and 3-isopropylmalate dehydratase (La01G00893), functioning in leucine biosynthesis (Sureshkumar et al., 2009; He et al., 2010). These changes correlated with the accumulation of isocitric acid and cis-aconitic acid, suggesting HT-induced activation of this metabolic pathway. Given that isocitric acid is synthesized from citric acid *via* aconitate hydratase, the coordinated upregulation of these enzymes supports their role in lavender’s heat response. However, the physiological significance of isocitric acid accumulation in plant thermotolerance remains unclear, representing an important area for future research to elucidate the molecular mechanisms underlying lavender’s HS adaptation. The C5-branched dibasic acid metabolism was another putatively enriched pathway (*p* < 0.05) in lavender under short-term HT stress, which aligned with trends observed in the transcriptome. It was characterized by the marked upregulation of 3-isopropylmalate dehydratase (La01G00893) and substantial accumulation of three key metabolites: cis-aconitic acid, trans-aconitic acid, and α-ketoglutaric acid. The accumulation of these metabolites suggests enhanced Krebs cycle activity, as both cis-aconitic acid and α-ketoglutaric acid are central intermediates in this cycle, while trans-aconitic acid likely forms from its cis-isomer through heat-induced isomerization. The significant increase in *α*-ketoglutaric acid is particularly noteworthy as it serves as a crucial precursor for glutamate biosynthesis. The upregulation of 3-isopropylmalate dehydratase, an enzyme involved in both leucine biosynthesis and methionine chain elongation during aliphatic glucosinolate formation (Knill et al., 2009), indicates a potential coordinated activation of amino acid biosynthesis pathways alongside the Krebs cycle. These metabolic changes collectively suggest that lavender responds to HTS by modulating multiple interconnected metabolic pathways, potentially to maintain energy homeostasis and provide precursors for stress-responsive compounds under thermal stress conditions. The KEGG pathway analysis revealed that cis-aconitic acid, trans-aconitic acid, and α-ketoglutaric acid are associated with the TCA cycle, indicating enrichment (*p* < 0.05) of this pathway in lavender under short-term HS, a finding strongly corroborated by its significant enrichment (adjusted *p* < 0.05) in the transcriptomic data. Transcriptomic data further supported this observation through the upregulation of aconitate hydratase (La26G01092), which catalyzes the citrate-to-isocitrate isomerization *via* cis-aconitate and has been linked to oxidative stress tolerance (Moeder et al., 2006), suggesting that short-term HS may simultaneously induce oxidative stress in lavender, alongside the upregulation of pyruvate dehydrogenase E1 (La20G02788), a key enzyme in the pyruvate-to-acetyl-CoA conversion, reinforcing the heat-induced activation of the TCA cycle, as well as the consistent upregulation of putative aconitate hydratase (La25G00706) across all HT treatments, collectively demonstrating that short-term HS robustly stimulates the TCA cycle in lavender as part of its metabolic adaptation to thermal stress. The glyoxylate and dicarboxylate metabolism pathway showed enrichment (*p* < 0.05) in short-term HT-treated lavender, where key DAMs including α-ketoglutaric acid, isocitric acid, cis-aconitic acid and succinic acid were identified, with L-glutamic acid and L-serine showing increased accumulation after prolonged HT treatment (5 days) while also being associated with sulfur metabolism. Corresponding upregulated genes in this pathway included La26G01092 and La25G00706, along with isocitrate lyase (ICL, La24G00501) which mediates the glyoxylate cycle bypass of TCA cycle by converting isocitrate to glyoxylate and succinate, a process critical for lipid metabolism as shown in *Arabidopsis* (Cornah et al., 2004; Eastmond et al., 2000); glyoxylate/hydroxypyruvate/pyruvate reductase (2KGR, La17G00688) that functions in L-tartrate biosynthesis and redox balance maintenance (Jia et al., 2019); Catalase (La20G00353) for hydrogen peroxide detoxification (Takeuchi et al., 1995); ribulose bisphosphate carboxylase small subunit (RuBisCO, La03G01655) potentially supporting photosynthetic carbon fixation under HS (Suh et al., 1987); and malate dehydrogenase (MDH, La04G03011 and La22G00268) likely involved in maintaining TCA cycle activity and redox homeostasis. Notably, this pathway was also significantly enriched (adjusted *p* < 0.05) in our transcriptomic analysis (Fig. 6), providing convergent multi-omics evidence for its activation under heat stress. These findings collectively indicate that lavender activates multiple protective mechanisms involving glyoxylate/dicarboxylate metabolism, TCA cycle intermediates, and associated enzymes to cope with short-term HT stress, with particular importance of succinic acid accumulation and the coordinated action of ICL, 2KGR, catalase, RuBisCO and MDH in mediating metabolic adaptation, although the specific role of succinic acid in plant thermotolerance requires further investigation.

Under long-term HT treatment (D5), key intermediates in lysine degradation including 2-aminoadipic acid, 2-oxoadipic acid, pipecolic acid and L-saccharopine were significantly upregulated in lavender. Recent evidence indicates these metabolites primarily derive from lysine catabolism. The hydrophobic yet neutral 2-aminoadipic acid participates in multiple enzymatic reactions, being convertible to 2-oxoadipic acid and L-glutamic acid through oxoglutaric acid interaction. Lysine-derived 2-oxoadipic acid production occurs *via* both saccharopine and pipecolic acid pathways in the cytosol, with subsequent mitochondrial conversion to glutaryl-CoA and acetyl-CoA. This suggests prolonged HT enhances lysine degradation to maintain TCA cycle activity. Transcriptomic analysis revealed upregulation of key genes in this process, including Histone-lysine N-methyltransferase (SUVR3, La12G01656) and CLF (La02G01795, La16G01250) which regulate gene silencing through histone methylation (Saleh et al., 2007; Liu et al., 2018), probable enoyl-CoA hydratase 1 (La17G01080) involved in β-oxidation (Yamada et al., 2015), and aldehyde dehydrogenase (La02G01469) (Kirch, Nair & Bartels, 2001), collectively demonstrating coordinated metabolic adaptation to HTS through lysine degradation. Our metabolomic analysis revealed enrichment of valine, leucine, and isoleucine biosynthesis pathways in lavender under three-day HT treatment, with 2-oxoadipic acid being the only significantly upregulated intermediate during prolonged HT stress. The elevated expression of alanine-glyoxylate aminotransferase 2 homolog 3 (AGXT2L3, La11G00309) at D3 coincided with these metabolic changes. Since 2-oxoadipic acid is catabolized into acetyl-CoA, and given that AGXT2L3 upregulation enhances pyruvate production (Liepman & Olsen, 2003), both of which serve as key substrates for the TCA cycle, these coordinated metabolic responses strongly suggest that repeated HT stress promotes TCA cycle activity in lavender as an adaptive mechanism to sustain energy metabolism under thermal stress conditions. In long-term HT-treated lavender, galactose metabolism was co-enriched in both metabolomic and transcriptomic data, with significant accumulation of raffinose and stachyose. Raffinose, hydrolyzed by D-galactosidase into D-galactose and sucrose, showed HT-induced accumulation consistent with *Arabidopsis* findings (Reichelt et al., 2023). Transcriptomic analysis revealed upregulation of key enzymes in the raffinose family oligosaccharides (RFOs) pathway, including galactinol synthase 1 (La11G00108) and 2 (La00G05016, La26G01854), known to enhance stress tolerance (Panikulangara et al., 2004; Nishizawa, Yabuta & Shigeoka, 2008). The induction of galactinol-sucrose galactosyltransferases (La01G00963, La21G00772, La04G02510, La02G00372) and stachyose synthase (La01G03276, La10G00311) further supported RFO biosynthesis (Peters et al., 2010; Peterbauer et al., 2002). Additionally, upregulated ADP-glucose phosphorylase (La04G01352) and alpha-galactosidase 1 (La07G02161) indicated enhanced carbohydrate mobilization (McCoy et al., 2006; Kim et al., 2002), while bifunctional UDP-glucose 4-epimerase (La11G01094) linked galactose metabolism with cell wall biosynthesis (Rösti et al., 2007). These coordinated responses highlight the importance of galactose metabolism in lavender’s HT adaptation. The metabolomic and transcriptomic analyses revealed co-enrichment of fatty acid biosynthesis pathways in lavender under long-term HT treatment, with significant accumulation of decanoic acid (capric acid) and caprylic acid. While these saturated fatty acids have been documented in other plant systems (Okoronkwo, Ajomiwe & Ike-Amadi, 2015; Yuan, Braune & Gröngröft, 2023), their potential roles in plant thermotolerance remain unexplored. Transcriptomic data showed consistent upregulation of enoyl-CoA delta isomerase 2 (La22G01388) across all HT treatments, which plays a critical role in the beta-oxidation of unsaturated fatty acids by isomerizing double bonds in various enoyl-CoA species (Goepfert et al., 2008), suggesting its importance in metabolic adaptation to thermal stress. These findings highlight the involvement of fatty acid metabolism in lavender’s response to prolonged HT conditions. Our multi-omics analysis revealed co-enrichment of starch and sucrose metabolism pathways (metabolomic *p* < 0.05; transcriptomic adjusted *p* < 0.05) in lavender under long-term HT stress. Metabolomic data showed substantial accumulation of GDP-alpha-D-glucose, a key glycosyl donor for cell wall biosynthesis, though its role in thermotolerance remains unexplored. Transcriptomic analysis identified upregulation of critical enzymes including: Beta-glucosidase (La00G01285) involved in xyloglucan degradation (Larsbrink et al., 2014); trehalase (La01G01680) for trehalose metabolism (Foster, Jenkinson & Talbot , 2003); hexokinase-2 (La14G00970) mediating sugar phosphorylation (Jang et al., 1997); sucrose-phosphate synthase 1 (La01G01856) for sucrose biosynthesis (Komatsu et al., 1996); and alpha,alpha-trehalose-phosphate synthase (La11G01799, La19G01538). We also observed significant upregulation of glucan endo-1,3-beta-glucosidase 4 (La15G01327, La04G01419), 4-alpha-glucanotransferase (La04G02575) involved in maltose metabolism (Chia et al., 2004), alpha-amylase 3 (La26G01053) for starch degradation (Seung et al., 2013), and alpha-1,4 glucan phosphorylase (La09G02167). These coordinated changes demonstrate a comprehensive metabolic reprogramming in lavender to cope with HT stress through enhanced carbohydrate metabolism and energy mobilization.

## Conclusions

This study elucidates the biphasic thermal adaptation mechanisms of *L. angustifolia* under HTS through integrated transcriptomic and metabolomic analyses, revealing distinct short-term (H1–H3, D1) and long-term (D3–D5) response strategies. The short-term phase featured rapid Ca^2+^-mediated signaling, heat-shock protein activation, transient oxidative stress (elevated MDA), and osmoprotectant accumulation (proline, raffinose-family oligosaccharides), with key metabolic pathways like 2-oxocarboxylic acid metabolism, the TCA cycle, and glyoxylate/dicarboxylate metabolism being prominently enriched alongside upregulation of genes such as aconitate hydratase and UDP-glycosyltransferase and accumulation of intermediates including isocitric acid and *α*-ketoglutaric acid. In contrast, the long-term phase involved systemic metabolic reorganization, including enhanced phenylpropanoid biosynthesis, fatty acid elongation (*e.g.*, capric acid), and restructuring of carbohydrate metabolism (*e.g.*, galactose and starch/sucrose pathways), supported by upregulated regulators like galactinol synthase and enoyl-CoA isomerase, which facilitated sustained thermotolerance through raffinose accumulation and lipid metabolism, while lysine degradation and amino acid metabolism maintained energy homeostasis under prolonged stress. These findings establish a comprehensive molecular framework for *L. angustifolia*’s thermotolerance, highlighting the transition from immediate protection to metabolic optimization and providing stage-specific targets for breeding climate-resilient cultivars, with direct implications for safeguarding medicinal crop production under global warming scenarios.

## Supplemental Information

10.7717/peerj.21294/supp-1 Supplemental Information 1 Supplementary Tables

10.7717/peerj.21294/supp-2 Supplemental Information 2 Checklist

10.7717/peerj.21294/supp-3 Supplemental Information 3 Supplementary figure

10.7717/peerj.21294/supp-4 Supplemental Information 4 Raw data for Fig. 1: MDA and proline (PRO) contents in *L. angustifolia* under different high-temperature stress treatments

10.7717/peerj.21294/supp-5 Supplemental Information 5 Raw data for Fig. 2: RNA-seq analysis including PCA, DEGs heatmap, statistical classification, and Venn diagram

10.7717/peerj.21294/supp-6 Supplemental Information 6 Raw data for Fig. 3: GO enrichment analysis of DEGs across all treatment comparisons

10.7717/peerj.21294/supp-7 Supplemental Information 7 Raw data for Fig. 4: KEGG pathway enrichment analysis of DEGs across all treatment comparisons

10.7717/peerj.21294/supp-8 Supplemental Information 8 Raw data for Fig. 5: Metabolomic profiling including PCA, Venn diagram of DAMs, and statistical distribution of metabolite classes

10.7717/peerj.21294/supp-9 Supplemental Information 9 Raw data for Fig. 6: Integrated pathway analysis showing overlapping KEGG pathways between transcriptomic and metabolomic datasets

10.7717/peerj.21294/supp-10 Supplemental Information 10 Raw data for Fig. 7: Heatmap of DEGs associated with top KEGG pathways under different stress conditions

10.7717/peerj.21294/supp-11 Supplemental Information 11 Raw data for Fig. 8: Heatmap of DAMs under different stress treatments

10.7717/peerj.21294/supp-12 Supplemental Information 12 Raw data for Fig. 9: qRT-PCR validation of selected DEGs, including RNA-seq and qRT-PCR expression values

## Additional Information and Declarations

### Data Availability

The following information was supplied regarding data availability: The raw data are available in the Supplemental Files. The transcriptomic sequencing data generated in this study are available in the National Center for Biotechnology Information (NCBI) Sequence Read Archive (SRA): PRJNA1268729 (SRA run accessions: SRR33730954–SRR33730977). The untargeted metabolomic data is available at the EMBL-EBI MetaboLights database: MTBLS12670.
