# Supplementary figures and images for: Integrated transcriptomic and metabolomic analyses reveal biphasic thermal adaptation strategies in Lavandula angustifolia under high-temperature stress
Source: PeerJ. 2026 Jun 17;14:e21294. doi: 10.7717/peerj.21294 (PMC13282949; doi:10.7717/peerj.21294)

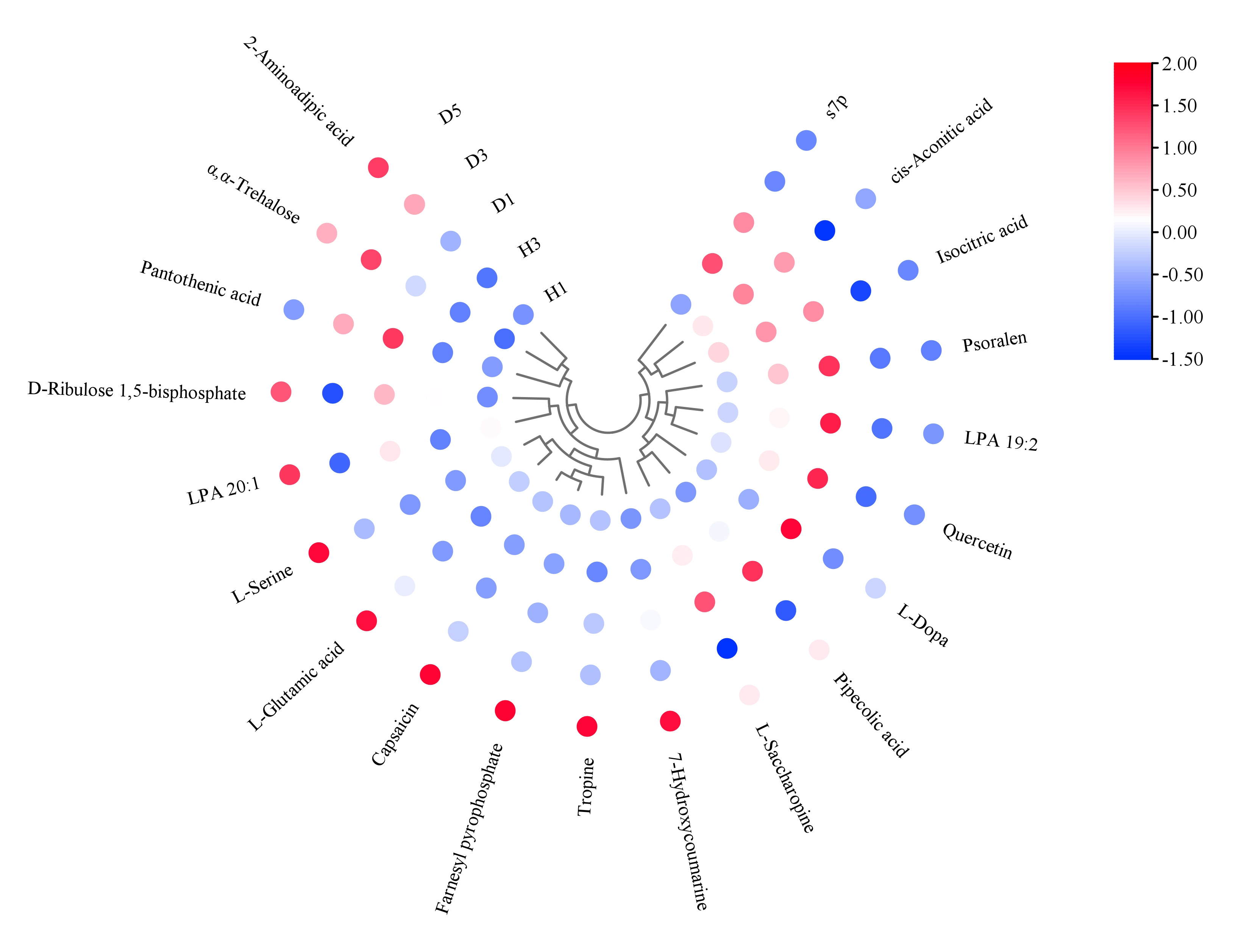

Supplement: Supplemental Information 3 [file peerj-14-21294-s003.jpg]
